# Apathy is associated with faster global cognitive decline and early nursing home admission in dementia with Lewy bodies

**DOI:** 10.1186/s13195-018-0416-5

**Published:** 2018-08-18

**Authors:** Monica H. Breitve, Kolbjørn Brønnick, Luiza J. Chwiszczuk, Minna J. Hynninen, Dag Aarsland, Arvid Rongve

**Affiliations:** 1 Department of Research and Innovation, Helse Fonna HF Haugesund Hospital, Postbox 2170, N-5504 Haugesund, Norway; 2 Department of Geriatric Psychiatry, Clinic of Psychiatry, Helse Fonna HF Haugesund Hospital, Postbox 2170, N-5504 Haugesund, Norway; 3 Faculty of Medicine, University of Bergen, Postbox 7804, 5020 Bergen, Norway; 4 TIPS – Centre for Clinical Research in Psychosis, Stavanger University Hospital, 4011 Stavanger, Norway; 5 Network for Medical Sciences, University of Stavanger, 4036 Stavanger, Norway; 6 Department of Clinical Psychology, University of Bergen, Christies gate 12, 5015 Bergen, Norway; 7 NKS Olaviken Gerontopsychiatric Hospital, Askvegen 150, 5306 Erdal, Norway; 8 Center for Age-Related Diseases (SESAM), Stavanger University Hospital, Armauer Hansens vei 20, 4011 Stavanger, Norway; 9 Department of Old Age Psychiatry, Institute of Psychiatry and Neuroscience, King’s College, London, UK

**Keywords:** Dementia, Alzheimer’s disease, Dementia with Lewy bodies, Apathy, Neuropsychology, Longitudinal, Survival

## Abstract

**Background:**

Little is known about the consequences of apathy in dementia with Lewy bodies (DLB), because previous research on apathy in dementia focused mainly on Alzheimer’s disease (AD).

**Methods:**

In this longitudinal study, we included patients with AD (*n* = 128) and patients with DLB (*n* = 81). At baseline, we analyzed the associations between apathy and cognition in the total sample and in AD and DLB separately. Generalized linear mixed models were used to investigate the association between apathy and Mini Mental State Examination (MMSE) over 4 years, and the Kaplan-Meier method was used to assess the association between apathy and survival or nursing home admission.

**Results:**

In patients with DLB, apathy was associated with a faster global cognitive decline (MMSE) over 4 years. Patients with DLB and apathy had shorter time until nursing home admission than DLB patients without apathy and patients with AD, regardless of apathy. At baseline, patients with apathy had decreased performance on the Stroop color test and a composite executive function score. Neurocognition was unaffected by apathy in AD, but DLB patients with apathy had more verbal learning difficulties.

**Conclusions:**

Apathy seems to be associated with more serious symptomatology in DLB than in AD. It is important to focus on apathy in dementia because it is one of the most prevalent and disturbing behavioral and psychological symptoms.

## Background

Apathy, defined as reduced motivation, reduced voluntary and goal-directed behavior, or reduced social interests and emotional blunting [1], is one of the most prevalent and important neuropsychiatric symptoms in dementia [2–4]. Reported apathy prevalence rates for outpatients with Alzheimer’s disease (AD) lie between 25% and 93%, and estimates exceed 50% for patients with mild dementia with Lewy bodies (DLB) [2, 5]. Apathy can be divided into cognitive, motor, and affective symptoms, depending on the most affected prefrontal-basal ganglia circuits. However, the neurobiology of apathy is still not well understood [4].

Symptoms seen in neuropsychiatric disorders can mask, mimic, or enhance apathy. Apathy is highly correlated with depression in patients with AD [6], and the two can be difficult to separate owing to overlapping symptoms. Nonetheless, apathy is a distinct syndrome from depression [7]. It is characterized by a lack of negative thoughts, less emotional distress, less sadness, less vegetative symptoms, and fewer somatic complaints than typically seen in depression [4].

Apathy in AD is associated with increased caregiver burden [8], decreased functioning in activities of daily living (ADL) [9], and more morbidity [10]. The presence of apathy in patients with mild cognitive impairment increases the risk of conversion to AD [11]. Previous findings regarding cognitive dysfunction in patients with apathy are contradictory. It has been reported that in AD, patients with apathy have lower Mini Mental State Examination (MMSE) scores than patients without apathy [12], whereas others have found no differences [13–15]. In studies that also included a small number of patients with DLB, no associations were found between apathy and MMSE scores [16, 17]. In neuropsychological tests, patients with AD and apathy have demonstrated more impairment in impulse control [18, 19], attention [18], visual memory [15], verbal memory [13, 18], naming, phonological verbal fluency [13], and semantic verbal fluency [14] than patients with AD without apathy. For patients with DLB, there were no associations between apathy and verbal memory, phonemic verbal fluency, and naming, except for a negative correlation between apathy and an executive task of sorting [17]. Patients with AD that, during a period of 1–4 years, developed apathy showed a faster cognitive and ADL decline than patients who did not develop apathy [7]. Even though an apathy-executive function syndrome has been described in different dementias, and although apathy rather than depression influences executive skills [20], the results of previous research are internally contradictory and inconclusive.

In extreme cases, apathy can be seen as a way of dying, because adequate response to changes in the environment is necessary for survival. The association between apathy and higher mortality is well documented in patients with AD [10, 21]. Apathy may also lead to faster nursing home admission for patients with AD [22].

There are few studies regarding apathy in AD and particularly few in DLB. We therefore investigated whether apathy was associated with shorter survival and higher risk of nursing home admission, as well as if apathy was associated with cognitive impairment at baseline. Finally, we examined if apathy was associated with faster global cognitive decline over 4 years as measured by MMSE. All analyses were performed in the total sample and stratified in AD and DLB.

## Methods

### Subjects

**Fig. 1:**
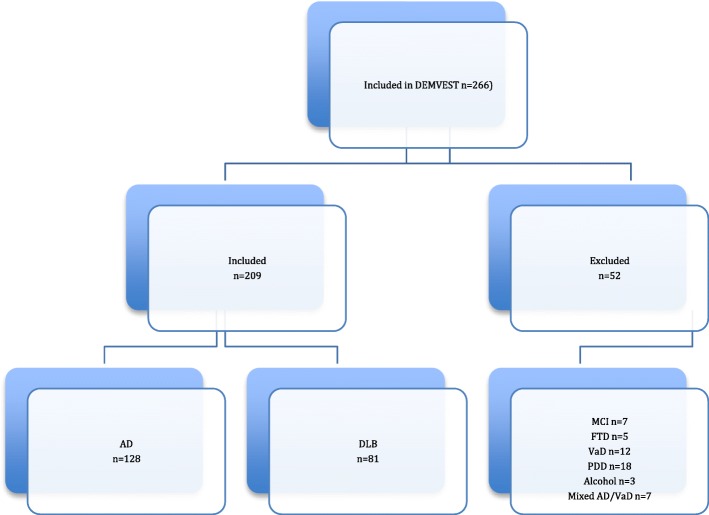
Study flowchart. *AD* Alzheimer’s disease, *DLB* Dementia with Lewy bodies, *FTD* Frontotemporal dementia, *MCI* Mild cognitive impairment, *VaD* Vascular dementia, PDD Parkinson’s disease dementia, *DEMVEST* Dementia Study of Western Norway

In a longitudinal dementia study in western Norway (DemVest), 266 outpatients with a first-time diagnosis of dementia (by MMSE [23] score of at least 20 or Clinical Dementia Rating [CDR] 1 [24]) at clinics of old age psychiatry or geriatric medicine were included and followed annually from 2005 (*see* Fig. 1). Acute delirium, confusion, terminal illness, bipolar or psychotic disorder, or recently diagnosed major somatic illness were causes for exclusion. The current study sample comprises patients diagnosed with AD (*n* = 128) and DLB (*n* = 81). The study was approved by the Regional Committee for Medical and Health Research Ethics in Western Norway and the Norwegian Social Science Data Services. Patients gave written consent to participate.

### Measures

#### Dementia diagnosis

At baseline, two independent raters set the dementia diagnoses on the basis of criteria of the *Diagnostic and Statistical Manual of Mental Disorders, Fourth Edition*. The diagnoses were revised by an expert team after 2 and 5 years, and the last revised diagnoses were used in the analyses. DLB diagnosis was made according to the revised consensus criteria of McKeith et al. [25], and AD diagnosis was made according to the criteria of the National Institute of Neurological and Communicative Disorders and Stroke-Alzheimer’s Disease and Related Disorders Association [26]. Cerebral magnetic resonance imaging or computed tomography was conducted in all patients to exclude other causes of dementia. Ioflupane single-photon emission computed tomography (^123^I-FP-CIT SPECT; DaTSCAN) was performed in 55 of the patients. Diagnoses were neuropathologically confirmed in a subsample.

#### Clinical and cognitive measures

Apathy, depression, and caregiver stress were rated by caregivers using the Neuropsychiatric Inventory (NPI) [27]. Symptoms were assessed for the last 30 days, in frequency (1–4) and severity (1–3), here applied both as a computed continuous variable (frequency × intensity) and as a dichotomized variable (clinically vs non-clinically significant), where a score above 3 was seen as clinically significant [5]. The Unified Parkinson’s Disease Rating Scale [28] was used for rating parkinsonism, and the patients’ general health status was measured by the Cumulative Illness Rating Scale (CIRS) [29]. The CDR [24] global score was used for measuring global dementia severity, and the MMSE [23] was used for cognitive screening. The 15-item Boston Naming Test [30] was used to measure naming. Executive functions were measured by the Stroop test [31], Controlled Oral Word Association Test (COWAT) semantic fluency [32], Trail Making Test A (TMT A) [33], and MMSE subtraction or backward spelling. Verbal learning and memory were measured with the California Verbal Learning Test, Second Edition (CVLT-II) [34], and MMSE delayed recall. Visuospatial function was measured by silhouettes and cubes on the Visual Object and Space Perception Battery [35] and MMSE pentagon. Raw scores were used, except when analyzing domains, in which case we used raw scores standardized into z-scores that were computed into neuropsychological domains. The raw scores on the TMT A were reversed for the analyses.

#### Statistical analyses

Statistical analyses were conducted with IBM SPSS Statistics version 23.0 software (IBM, Armonk, NY, USA). Differences at baseline were analyzed with the *t* test for normal distributions, the Mann-Whitney *U* test for nonnormally distributed data, and the Pearson’s chi-square test for categorical data. Univariate analysis of variance was used to control for depression. Analyses were performed on the total sample and stratified into AD and DLB. Survival analysis was done with the Kaplan-Meier method, and Cox regression was used to control for age at inclusion, depression, and caregiver stress. Generalized linear mixed models (GLMMs) were used for assessing the association between apathy and MMSE scores over time (apathy × time × MMSE), with the confounders sex, age, education, and depression entered as covariates. Subjects were set as random effects to correct for dependency of repeated measures, using a first-order autoregressive structure with homogeneous variance and gamma distribution with a log link.

## Results

### Baseline analysis

In the total sample, when comparing patients with apathy and those without apathy, we found a lower percentage of patients with AD, fewer women, and fewer patients on dementia medication among patients with apathy. Patients with apathy had higher scores on the CDR global, NPI depression scale, and CIRS (*see* Table 1). In neuropsychological tests, patients with apathy had decreased performance on the Stroop color test and COWAT animals, as well as lower computed scores on tests measuring executive functions. After controlling for depression, the COWAT animals test was no longer significant (*see* Table 2). When stratifying the analyses according to dementia diagnosis, we observed that patients with AD and apathy had higher scores on the CDR global score, meaning more advanced dementia, and the NPI depression scale (i.e., more depressive symptoms reported by caregivers). There were no significant differences in neuropsychological tests between patients with and without apathy, and the difference in the executive domain also disappeared. Patients with DLB and apathy had higher depression scores and decreased learning on CVLT-II List A total, even after controlling for depression, than patients with DLB without apathy (*see* Table 2).

**Table 1:** Characteristics of patients with and without apathy at baseline

	Total sample			AD			DLB		
	Nonapathy (*n* = 124)	Apathy (*n* = 72)	*p* Value*	Nonapathy (*n* = 85)	Apathy (*n* = 35)	*p* Value*	Nonapathy (*n* = 39)	Apathy (*n* = 37)	*p* Value*
AD/DLB (%)	68.5/31.5	48.6/51.4	.006						
Sex, male/female	39/85	33/39	.044	64/21	22/13	.169	21/18	17/20	.491
Age, years (SD)	76.2 (7.2)	75.8 (8.6)	.702	75.7 (7.7)	75.7 (7.8)	.865	77.3 (5.9)	75.9 (8.2)	.382
Education, years (SD)	9.8 (3.1)	9.3 (2.6)	.311	9.7 (3.1)	9.2 (2.3)	.497	9.9 (3.0)	9.4 (2.9)	.400
CDR global (IQR)	0.8 (0.5)	1.0 (0.0)	<.001	0.7 (0.5)	1.0 (0.0)	<.001	0.9 (0.5)	1.1 (0.4)	.072
Depression, NPI (SD)	1.4 (1.9)	3.2 (3.1)	<.001	1.4 (1.9)	3.2 (3.1)	.001	1.3 (1.8)	3.2 (3.0)	.003
UPDRS (SD)	6.4 (10.6)	7.6 (10.4)	.175	1.5 (2.6)	2.5 (4.0)	.296	15.9 (13.6)	12.4 (12.2)	.245
CIRS (SD)	5.5 (2.4)	6.3 (2.7)	.034	5.1 (2.1)	5.7 (2.5)	.244	6.5 (2.9)	6.8 (2.8)	.641
Duration of dementia symptoms before baseline, months (SD)	34.8 (21.9)	40.8 (24.7)	.104	32.4 (21.3)	36.6 (22.5)	.298	40.0 (22.4)	44.9 (26.4)	.396
Dementia medication at baseline, yes (%)	52.7	35.8	.029	51.9	41.2	.296	54.8	30.3	.047
Dementia medication at follow-up 1, yes (%)	74.5	79.6	.477	71.6	80.0	.384	81.5	79.2	.835

*Abbreviations: AD* Alzheimer’s disease, *DLB* Dementia with Lewy bodies, *CDR* Clinical Dementia Rating, *CIRS* Cumulative Illness Rating Scale, *UPDRS* Unified Parkinson’s Disease Rating Scale

* Differences were analyzed using the Mann-Whitney *U* test and Pearson’s chi-square test

**Table 2:** Neuropsychological test scores at baseline in patients with and without apathy

	Total sample			AD			DLB		
	Nonapathy (*n* = 124)	Apathy (*n* = 72)	*p* Value*	Nonapathy (*n* = 85)	Apathy (*n* = 35)	*p* value*	Nonapathy (*n* = 39)	Apathy (*n* = 37)	*p* Value*
MMSE total (SD)	23.6 (2.6)	23.4 (2.8)	.415	23.7 (2.5)	23.6 (2.2)	.486	23.4 (3.0)	23.11(3.3)	.736
MMSE pentagon, correct copy (%)	61.8	51.4	.102	70.9	61.8	.340	48.4	45.5	.814
MMSE subtraction/word (SD)	3.7 (1.4)	3.6 (1.6)	.973	4.0 (1.3)	4.0 (1.4)	.593	3.2 (1.5)	3.3 (1.8)	.862
MMSE memory (SD)	1.0 (1.0)	1.0 (1.0)	.886	0.8 (0.9)	0.6 (0.8)	.462	1.4 (0.9)	1.4 (1.1)	.870
CVLT-II List A total (SD)	20.4 (7.5)	18.8 (9.2)	.092	18.5 (6.4)	18.6 (7.7)	.695	24.5 (8.3)	19.1 (10.5)	.020
CVLT-II delayed recall (SD)	1.9 (2.4)	1.6 (2.2)	.416	1.4 (1.9)	1.2 (2.1)	.496	3.1 (2.8)	2.1 (2.3)	.141
VOSP silhouettes (SD)	13.7 (5.0)	12.9 (5.2)	.269	13.8 (5.2)	13.0 (6.2)	.480	13.7 (4.6)	12.8 (4.2)	.084
VOSP cubes (SD)	6.4 (3.1)	6.5 (3.0)	.885	7.2 (2.3)	7.4 (2.3)	.711	5.2 (3.7)	5.6 (3.5)	.652
BNT (SD)	10.1 (2.9)	10.8 (2.9)	.165	10.1 (3.1)	10.4 (3.2)	.644	10.1 (2.4)	11.1 (2.5)	.146
Stroop word (SD)	69.4 (22.9)	62.6 (20.6)	.087	75.9 (22.2)	70.3 (18.3)	.260	55.1 (17.4)	54.4 (20.1)	.111
Stroop color (SD)	44.0 (15.6)	38.5 (14.5)	.021	47.4 (15.6)	43.0 (11.6)	.148	36.3 (12.5)	33.7 (15.9)	.302
Stroop color-word (SD)	16.2 (0.9)	13.4 (8.3)	.133	18.3 (11.5)	13.9 (8.9)	.103	11.5 (7.3)	13.0 (7.7)	.405
COWAT animals (SD)	12.2 (4.4)	10.7 (4.4)	.014	12.3 (4.2)	11.2 (4.4)	.146	12.0 (4.7)	10.3 (4.4)	.108
Trail Making Test A (SD)	111.0 (69.0)	124.2 (92.1)	.686	96.8 (63.7)	113.4 (82.1)	.544	143.1 (70.3)	136.1 (101.9)	.173
Memory domain, z-scores (SD)	0.03 (0.8)	−0.04 (0.9)	.395	−0.17 (0.87)	−0.25 (0.8)	.422	0.48 (0.8)	0.15 (0.9)	.152
Visuospatial domain, z-scores (SD)	0.06 (0.7)	−0.09 (0.7)	.132	0.17 (0.7)	0.06 (0.8)	.406	−0.17 (0.8)	−0.22 (0.69)	.652
Executive domain, z-scores (SD)	0.08 (0.7)	−0.18 (0.8)	.014	0.30 (0.6)	0.05 (0.6)	.104	−0.30 (0.6)	−0.39 (0.8)	.823

*Abbreviations: AD* Alzheimer’s disease, *BNT* Boston Naming Test, *COWAT* Controlled Oral Word Association Test, *CVLT-II* California Verbal Learning Test, Second Edition, *DLB* Dementia with Lewy bodies, *MMSE* Mini Mental State Examination, *VOSP* Visual Object and Space Perception Battery

*Differences were analyzed using the Mann-Whitney *U* test and Pearson’s chi-square test

### Longitudinal analysis

**Fig. 2:**
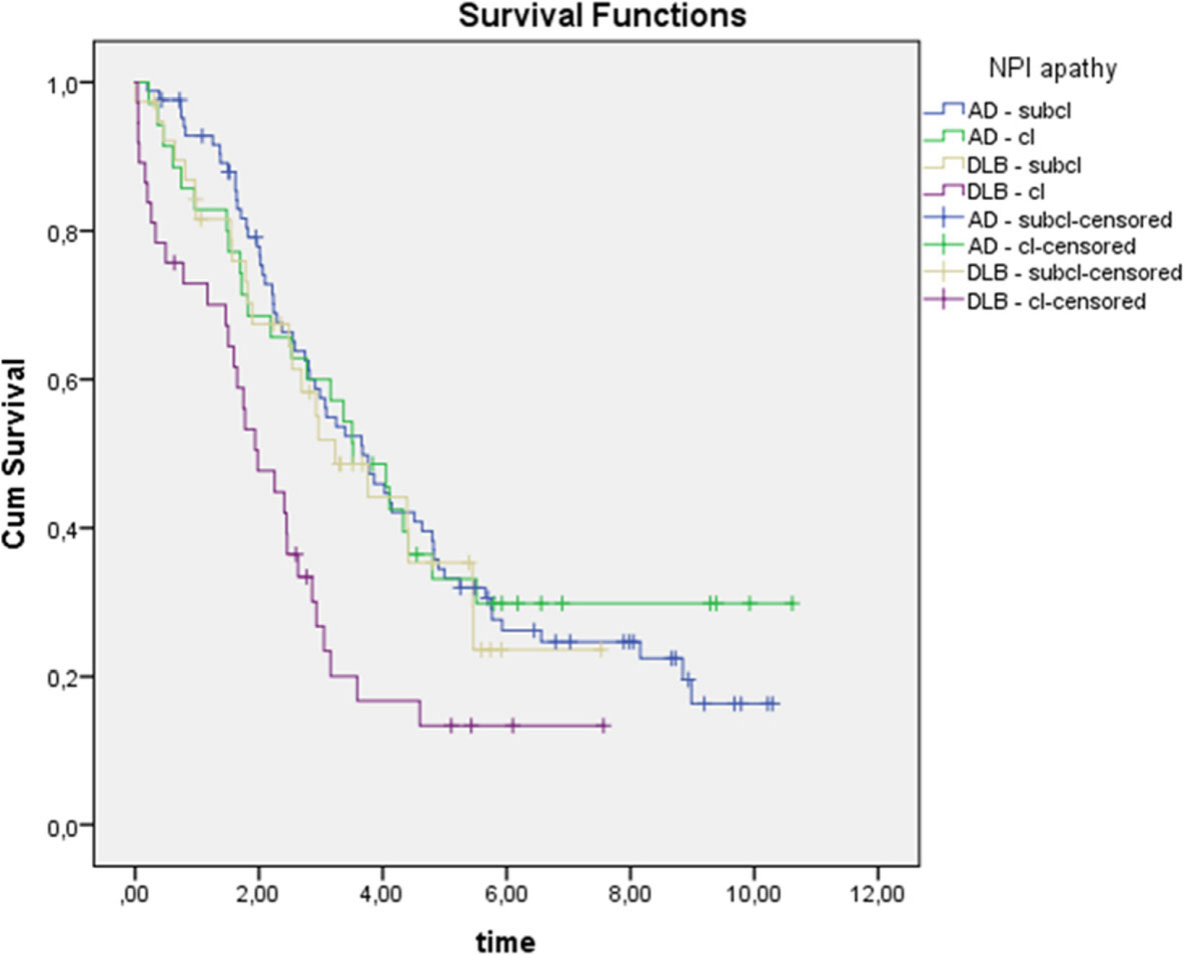
Time until nursing home admission. *AD* Alzheimer’s disease, *DLB* Dementia with Lewy bodies

In patients with DLB, there was a statistically significant association between apathy and decline in MMSE scores over 4 years (main effect *F* = 2.045, *p* = .006). There were no significant differences in the total sample or in the AD group. At baseline, apathy was not associated with survival or nursing home admission for the total sample. Patients with DLB lived shorter than patients with AD (*p* ≤ .001) (median 4.0/4.6 vs 7.1/7.2 years), but there was no significant difference between patients with or without clinically significant apathy. Patients with DLB and clinically significant apathy had a shorter time until nursing home admission than patients with DLB and no apathy, or than patients with AD, regardless of apathy (*p* = .001) (median 2.0 vs 3.2/3.5/3.7 years) (*see* Fig. 2).

## Discussion

Patients with dementia and apathy had reduced executive function as measured in neuropsychological domains and in the Stroop color test, even after controlling for depression, which was moderately correlated with apathy. When comparing AD and DLB separately, the only significant difference was found in the DLB group, where patients with apathy had more verbal learning deficits than the nonapathy group. This could be explained by patients with AD having temporal lobe deficits and difficulties with learning in general, regardless of frontal lobe function/apathy. In DLB where temporal lobe pathology is not advanced, patients with apathy and frontal lobe dysfunction/apathy have deficits in using learning strategies that are controlled by the frontal lobes and have scores at the same level as patients with AD. However, we found no significant differences in the executive domain in the DLB group, which then would have been expected. In the executive domain, we included a test typically associated with attention. The relationship between attention and executive functions is still debated. It has been suggested that they are interrelated but have different anatomical components [36], so the use of a too-wide concept for the executive domain and too few or suboptimal executive tests (i.e., semantic fluency instead of phonological fluency) could explain the internal contradictory result. There was also more use of dementia medication in the nonapathy DLB group, which could have a positive effect on apathy and cognition. In addition, multiple analyses were performed, so there is a risk of committing family-wise statistical errors (inflated risk of type I errors). It should also be noted, as previously mentioned, that other studies have found worse cognitive performance in AD patients with apathy than in patients without apathy [13–15, 18, 19].

In sum, the results are not as expected, because in general we found few significant associations with apathy for neuropsychological tests at baseline. On the basis of previous studies, including studies by our group, there is a lack of strong support for the apathy-dysexecutive-frontal syndrome in cognitive tests, whereas it is clearly supported in brain function studies with different approaches [37]. There are only a few studies regarding apathy and neuropsychological tests, and the numbers of patients are often small; therefore, unexpected nonsignificant results could be due to low statistical power. To the best of our knowledge of this literature, we have included the largest number of patients with DLB and the third largest number of patients with AD.

In patients with DLB, we found that apathy was associated with a faster progression in MMSE over 4 years, and it was not explained by depression. Neither is it likely to be caused by the use of dementia medication, because the same number of patients used these drugs after the day of inclusion. The observation may be a result of more negative interactions between apathy and other Behavioral and Psychological Symptoms of Dementia (BPSD), such as visual hallucinations, that are more prevalent in DLB than in AD, which also are correlated with executive dysfunction [38]. It could also be due to differences in genetic profiles and cortical pathology. If dementia medication had a hypothetical positive effect on apathy and progression, this effect would have had its origin from before the patients were included in the study.

Patients with DLB had shorter survival time than patients with AD, but this was not associated with apathy. Several previous studies have also indicated a shorter survival in DLB than in AD [39, 40]. On one hand, time until nursing home admission was more than 1 year shorter for DLB with apathy than for those without, but on the other hand, we found the same rate of survival for the two groups. If we put forward the premise that patients, at group levels, are expected to live approximately the same period of time after nursing home admission, we could hypothetically explain the equal time of survival by better caregiving, more activity, and treatment at the nursing home. This counteracts apathy and helps patients with DLB and apathy live as long as patients with DLB without apathy. There were no statistical differences in health status for patients with and without apathy in DLB, and therefore this could not explain the observation. We do not have information about the quality of caregiver support, but caregiver stress did not influence the results.

Some caution should be taken when interpreting the results. There were some missing data, because a natural consequence of studying patients who are elderly and have a progressing disease. GLMM analyses are an appropriate approach that can compensate to some degree for missing data at random.

As anyone who has performed neuropsychological testing of apathetic patients knows, these patients’ lack of motivation to perform on tests is an obvious problem. We do not have information about the patients’ test effort and therefore could not control for it in the analyses. We have not discussed different types of apathy or the concept of apathy and differential symptoms/diagnosis, but we have operationalized it as what is measured by the apathy subscale on the NPI, rated by the caregivers. Neither did we include information from the patients, because apathy and insight are negatively correlated and will affect validity [4]. Some have asked for a unified assessment tool for apathy in the dementia population. The NPI is one of the most often used instruments for assessing BPSD in patients with dementia and should adequately assess apathy and depression independently [41], but it should be noted that only few studies have investigated the psychometric properties of the NPI in the DLB population.

The MMSE, which was used in the longitudinal analyses, is also often criticized for not having high sensitivity for changes in cognition in pure DLB [42]. However, the test has been found to be valid in patients with Parkinson’s disease, who share similar pathology to patients with DLB [43].

In this study, we performed multiple analyses at baseline, and there is a legitimate possibility of observing significant results simply due to chance. However, by using methods such as the Bonferroni correction, we could risk rejecting H1 even if it were true (type II error). Because we mainly had negative results, and because the consequence of falsely stating that apathy has no negative associations could be worse than the opposite, we chose not to adjust the alpha-level. Nevertheless, we acknowledge that an analytic approach defined purely a priori would have yielded more robust results.

A strength of the study is that it provides knowledge about apathy in DLB, which is an issue that has not been addressed before and can offer useful information to patients, their caregivers, clinicians, and the community. It provides information on prognosis and focuses on apathy in dementia, which should be assessed and intervened when necessary. The study also has a relatively large number of patients with DLB, and they were followed over 4 years with regard to cognition, and longer for nursing home admission and survival. Patients were thoroughly evaluated and diagnosed (i.e., the majority of patients with DLB had undergone ^123^I-FP-CIT SPECT [DaTSCAN]), including brain autopsy. We have previously reported that in our sample, autopsy in 43 cases showed 92% sensitivity and 83% specificity for the clinical diagnoses [44].

## Conclusions

Apathy seems to be associated with more serious consequences in DLB than in AD. Although there were only few differences in neuropsychological tests at baseline, apathy in patients with DLB was associated with faster cognitive decline in MMSE over 4 years and shorter time until nursing home admission. It is important to focus on apathy in dementia because it is one of the most prevalent BPSD symptoms, and interventions are required when apathy is present. More research is needed (i.e., studies with more patients with DLB) that differentiates apathy into subcategories and includes genetic information and pathologically confirmed diagnosis.

## Abbreviations

^123^I-FP-CIT SPECT: Ioflupane single-photon emission computed tomography
AD: Alzheimer’s disease
ADL: Activities of daily living
BNT: Boston Naming Test
BPSD: Behavioral and Psychological Symptoms of Dementia
CDR: Clinical Dementia Rating
CIRS: Cumulative Illness Rating Scale
COWAT: Controlled Oral Word Association Test
CVLT-II: California Verbal Learning Test, Second Edition
DEMVEST: Dementia Study of Western Norway
DLB: Dementia with Lewy bodies
FTD: Frontotemporal dementia
MCI: Mild cognitive impairment
MMSE: Mini Mental State Examination
NPI: Neuropsychiatric Inventory
PDD: Parkinson’s disease dementia
TMT A: Trail Making Test A
UPDRS: Unified Parkinson’s Disease Rating Scale
VaD: Vascular dementia
VOSP: Visual Object and Space Perception Battery
